# Perioperative adjuvant corticosteroids for post-operative analgesia in elective knee surgery – A systematic review

**DOI:** 10.1186/s13643-017-0485-8

**Published:** 2017-04-27

**Authors:** Hasan Raza Mohammad, Marialena Trivella, Thomas W. Hamilton, Louise Strickland, David Murray, Hemant Pandit

**Affiliations:** 1 Nuffield Department of Orthopaedics, Rheumatology and Musculoskeletal Sciences (NDORMS), University of Oxford, Oxford, UK; 2 Centre for Statistics in Medicine, University of Oxford, Oxford, UK; 3 Leeds Institute of Rheumatic and Musculoskeletal Medicine, University of Leeds, Chapel Allerton Hospital, Chapeltown Road, Leeds, LS7 4SA UK

**Keywords:** Analgesia, Corticosteroids, Knee, Perioperative, Surgery

## Abstract

**Background:**

Elective knee surgery is performed to reduce chronic pain and improve function in degenerate knees. Treatment of acute post-operative pain is suboptimal in 75% of patients despite multimodal analgesic approaches resulting in higher post-operative opiate consumption. The effect of corticosteroids as an adjunct for post-operative pain control remains undefined.

**Methods:**

The databases MEDLINE, EMBASE and CENTRAL (Cochrane library) will be searched from their inception to present using broad search criteria for eligible randomised/quasi-randomised controlled trials investigating perioperative corticosteroid adjunctive use in elective knee surgery. Meta-analyses will be conducted according to the recommendations from the Cochrane Handbook for Systematic Reviews of Interventions.

**Discussion:**

This systematic review of the perioperative adjunctive use of corticosteroids will assess the analgesic effects, post-operative nausea and vomiting, opiate consumption, infection rates and time till discharge and assess whether adjunctive corticosteroids should be encouraged in elective knee surgery.

**Systematic review registration:**

PROPSERO CRD42016049336

**Electronic supplementary material:**

The online version of this article (doi:10.1186/s13643-017-0485-8) contains supplementary material, which is available to authorized users.

## Background

### Description of the condition

Treatment of acute post-operative pain following elective surgery is suboptimal with reports of up to 75% of patients having inadequate pain relief in the immediate post-operative period [1, 2].

Elective knee surgery is commonly performed to reduce chronic pain and improve function in degenerate knees. Approximately 160,000 knee and hip replacements are conducted annually with demand on the rise [3]. This is evidenced by the greater numbers of patients having to wait beyond the 18-week target for elective surgery. In 2014, 51,388 patients waited excess of this period compared to 92,739 in 2015 [4].

Effective analgesia is therefore paramount to early rehabilitation, deconditioning prevention and timely discharge to free up resources. By the year 2030, the demand for total knee arthroplasty alone is predicted to grow sixfold placing emphasis on post-operative pain optimization as a priority [5].

Traditionally, pain control following surgery heavily relied on oral opiates and patient-controlled analgesia. However unfortunately, opiates are associated with various side effects, some of which include respiratory depression, confusion, sedation, vomiting, pruritus and urinary retention with evidence suggesting longer hospital stays and higher socioeconomic costs. Furthermore, the light sedation, muscle weakness and dizziness opiates cause may have an impact on post-operative rehabilitation delaying it or even predisposing to increased risk of injuries [6, 7].

There is also evidence that optimization of immediate post-operative analgesia reduces chronic post-surgical pain occurrences and improves functional outcomes significantly [8].

### Description of the intervention

The limitations of opiate therapy combined with a deepening understanding of pain physiology and pathophysiology has been paramount to developing alternative pain management strategies.

Kehlet and Dahl [9] conceptualised the multimodal analgesia model, in which a combination of different analgesic classes used simultaneously act on different pathways but in effect complement each other thereby potentiating their analgesic effect. This in turn reduces the reliance on opiates. Common medications used in a multimodal approach include paracetamol, non-steroidal anti-inflammatory drugs and gabapentinoids [9].

The effectiveness of adjunctive agents to the multimodal approach have been studied extensively for medications including ketamine, gabapentin and paracetamol demonstrating clear benefits in reducing post-operative pain and opiate consumption. As a result, these medications have become incorporated into most multimodal approaches [10–12].

Corticosteroids are commonly used for post-operative nausea and vomiting and may have a role in post-operative pain control to further optimise current approaches [13]. Often, randomised controlled studies report conflicting results regarding their usage, and therefore, corticosteroids effect on post-operative pain remains undefined [14].

Corticosteroids have recently been used to reduce inflammation and damage to tissues in several conditions including rheumatoid arthritis with early studies in the dental field suggesting a role in reducing post-operative pain and oedema [15].

Unfortunately, corticosteroids are not without their side effects with reports of increased risk of infections, osteoporosis, wound dehiscence and muscle weakness [16]. Although these side effects usually refer to long-term steroid usage (>3 weeks) and not in a perioperative setting, their side effects must be monitored and the lowest effective dosage utilised.

### How the intervention might work

Corticosteroids can be divided into two main groups; glucocorticoids and mineralocorticoids. Mineralocorticoids are involved in electrolyte and water balance and are not utilised in multimodal pain management postoperatively. Therefore, when referring to corticosteroids in our review, our emphasis is placed on the glucocorticoid type.

During surgery, tissue injury initiates the release of proinflammatory cytokines and prostaglandins that cascade a pathway hypersensitising nociceptors thereby generating pain.

It is believed that all glucocorticoids bind to specific cytoplasmic glucocorticoid receptors thereby inhibiting prostaglandins normally released in tissue injury and hence the hypersensitisation process [17, 18]. Although glucocorticoids are well known medications, their precise mechanism of action remains unclear.

Furthermore, steroid hormone receptors are found throughout the central and peripheral nervous systems allowing them to assist regulate the growth, maturation and differentiation of neurones. This suggests their role is pivotal in pain perception [19]. Specifically, corticosteroids have been proved to reduce the spontaneous discharge from damaged nerves thereby reducing pain [17].

### Why is it important to do this review

Steroids have been utilised extensively for inflammatory conditions, immunomodulation for organ transplant and antiemetic properties in the immediate post-operative period. In surgery, they are often utilised as an adjunct in multimodal analgesic regimens although their effect on post-operative pain remains undefined. It is also important to ensure that corticosteroid administration is safe by monitoring their side effects such as infection and poor wound healing [15].

A review in this field would allow us to conclude whether or not adjunctive corticosteroid use has any effect on immediate post-operative pain. The results of our review will inform us of whether steroids should be incorporated into multimodal analgesic regimens for elective knee surgery and would have implications for elective surgery in general.

## Objectives

The objective of this review was to assess the effect of perioperative corticosteroids used as an adjuvant compared to those without corticosteroids in patients aged 18 and over undergoing elective knee surgery, while taking into consideration the route of administration.

## Methods/design

### Study registration

The protocol is registered on PROPSERO with CRD 42016049336 and follows the Preferred Reporting Items for Systematic Reviews and Meta-Analyses Protocol 2015 (Additional file 1).

### Criteria for considering studies for this review

#### Types of studies

We will include all randomised and quasi-randomised controlled trials, including cluster trials if they have a minimum of two comparison groups, one of which includes adjunct corticosteroid usage. Studies will be included irrespective of publication status or language.

#### Types of participants

We will include all trials with patients over the age of 18 and older undergoing elective knee surgery with no restriction on co-morbidities or ethnicity. Our definition of elective knee surgery encompasses all types of knee surgery, which includes total/partial knee arthroplasty, knee arthroscopy, ligament reconstruction, meniscal resection/repair and cartilage repair.

#### Types of interventions

Our definition of elective knee surgery is to encompass all types of knee surgery which will include total/partial knee arthroplasty, knee arthroscopy, ligament reconstruction, meniscal resection/repair and cartilage repair. We anticipate from our preliminary searches the vast majority of studies investigating steroids in knee surgery will fall broadly under arthroplasty or arthroscopy (given most ligament repairs, meniscal resections and cartilage are performed arthroscopically).

We will include all knee surgery procedures randomised controlled trials comparing the effect of corticosteroid administration perioperatively as an additional medication to standard analgesic therapy. Acceptable routes of corticosteroid administration will include oral, intravenous or intraarticular administration. Studies reporting on preoperative, intraoperative and post-operative administration will be included provided the corticosteroid is administered within 24 h prior to the procedure and no later than 24 h after surgery.

We will exclude papers administering steroids through neurones/epidurals as these are user dependant and can potentially damage nerves thereby confounding any effect of steroid administration on pain.

#### Types of outcome measures

i) Primary outcomes
  Mean pain score assessed on a pain scale at rest at 24 h following surgery. All pain scales reported by the various studies will be considered.
ii) Secondary outcomes
  - Mean pain score
    Mean pain score at rest assessed on a pain scale at rest at 12, 48 and 72 h postoperatively. All pain scales will be considered.
  - Opiate consumption
    Mean opiate consumption in oral morphine equivalents taken postoperatively over the first 24 h.
  - Nausea and vomiting
    The risk of post-operative nausea and vomiting (PONV) in the first 24 h postoperatively. We will measure this as binary data. We will also describe narratively the number of other gastrointestinal complications including ulcers and GI bleeding.
  - Infection
    The risk of infection during patient’s hospital stay postoperatively. We will measure this as binary data. We will narratively discuss the numbers of superficial and deep infections if reported by studies.
  - Time till discharge
    The time till discharge postoperatively.
  - Other adverse effects
    Other adverse effects not falling into outcomes stated. This would include delayed wound healing, glucose levels if measured, diabetes incidence and anything else reported in the literature.

### Electronic database searches

The following databases will be searched from their conception to 10 October 2016

1. PUBMED/OVID MEDLINE
2. EMBASE
3. CENTRAL (Cochrane library)

Appropriate medical subject headings or their equivalents were utilised for all databases searched using broad search criteria to increase sensitivity of the search. The broad fields included in the search will include surgery, corticosteroids and pain along with all variants of the terms. Additionally, the randomised controlled trial (RCT) term was utilised in our search. The search strategy was approved by an Oxford University Bodleian Librarian. The search strategies for each database are highlighted in Appendix 1.

### Searching for other resources

Extracted paper bibliographies will be searched for any articles that meet our inclusion criteria as well as by observing other review articles in the field.

### Data collection and analysis

#### Selection of studies

Studies collected from the search conducted will be screened independently for inclusion by two authors (HRM and TH) initially by title and abstract. Shortlisted papers will have complete manuscripts obtained and assessed using the eligibility criteria by (HRM and LS). Any disagreements will be initially discussed between HRM and LS and if no consensus reached by the involvement of a third author (MT). If needs be, the senior authors (DM, HP) will also be consulted.

The summary of the number of papers form search results and at each step will be summarised in a PRISMA flowchart. The shortlisted papers will then be screened for any duplicate data and be processed appropriately as per next section.

#### Data extraction and management

Data from the database search will be managed using EndNote software. Results from the original search on all databases will be merged and duplicates removed. All papers will have their abstracts and titles read by two authors (HM and TM) for shortlisting. Full papers will be read if further details are required or there is disagreement between the authors. If there is still disagreement, a third author (HP) will be consulted.

Data extraction from shortlisted papers will be conducted independently by two authors (HM and TH) and recorded onto a standardised Microsoft excel database with outcomes of interest. A third author (HP) will be contacted if any inconsistency in data collection is observed between the two authors. If additional information is required for any of the included studies, the corresponding authors for the papers will be contacted by email.

From each paper, the following information will be extracted: author, country, year of study, procedure type, type of anaesthesia, number of patients in each study arm, treatment in each study arm, route and dosage of steroid administration, all primary and secondary outcomes as previously described with their standard deviation (SD), confidence interval (CI) or standard error (SE).

#### Assessment of risk of bias in included studies

Two review authors (HM and TH) will independently assess the risk of bias (ROB) using the Cochrane Risk of bias tool [20]. The studies will be assessed as ‘high’, ‘low’, or ‘unclear’ risk of bias, and any judgement will be supported and reported, based on the information provided in the studies. Consensus on the judgements will be reached through discussion, and where necessary, a third review author will be consulted.

The following domains will be assessed for each included study

- Random sequence generation
- Allocation concealment
- Blinding
- Incomplete outcome data
- Selective outcome reporting
- Other bias

#### Measures of treatment effect

The dosage of steroids administered will be converted into oral prednisolone equivalents.

Outcomes of pain scores will be treated as continuous variables. If studies report different pain scales, we will explore whether widely accepted transformation techniques are available for obtaining the equivalent (0-10) VAS scores. If this is not possible, we will include these data and opt for the standardised mean difference when carrying out meta-analysis.

For continuous outcomes (time till discharge, opiate consumption in morphine equivalents, and pain scores) using the same measurement scales, we will record the mean, standard deviation, and total number of participants in the treatment and control groups, and will express the treatment effect as the mean and 95% CI. If outcomes are reported in different scales, we will use the standardised mean difference (SMD).

For binary outcomes (post-operative nausea and vomiting and infection), the risk ratio (RR) and 95% CI will be calculated. If small numbers of events are reported (<5% of the sample in each group), and the treatment groups are balanced, we will report the Peto’s odds ratio (Peto-OR) and 95% CI.

#### Unit of analysis issues

For outcomes pain at 12/24/48/72 h and infection, our unit of analysis will be number of knees. For outcomes nausea and vomiting, time till discharge, opiate consumption and other adverse effects, unit of analysis will be number of patients.

For studies involving multiple arms, where it is not possible to combine arms, they will be analysed by dividing either (i) the number of events and group size (for dichotomous) or (ii) the group size (for continuous), in the number of arms in the trial as appropriate.

If the search identifies eligible cluster trials, we will obtain the interclass correlation (ICC) and will include the cluster trials in the meta-analysis using the generic inverse variance method. If ICC is not available, we will borrow an appropriate ICC from similar trials.

We are not expecting any cross-over trials as this would be an inappropriate design in this case.

#### Dealing with missing data

Study authors will be contacted where possible to recover as much missing information as possible. Reasons for missing data will be explored through sensitivity analysis.

Where data is demonstrated graphically with no numerical description in the text, and if contact with study authors is not possible, Measure C software will be utilised to extrapolate the values from enlarged images of the graphs to the nearest pixel [21]

We will not utilise multiple imputation given its controversies for multiple scoring systems and potential small sample sizes in some of the studies included [2, 22].

Where data are not suitable for incorporation into meta-analysis, information will be summarised as a narrative or in tables, as appropriate.

#### Assessment of heterogeneity

We expect heterogeneity from measurement scales used for our outcomes of interest and different types of knee surgery procedures, and hence, we will be using the random effects model in all meta-analyses.

We will explore heterogeneity using the *I* ^2^ statistic and classify it as substantial if *I* ^2^ > 90%. Pooled results with heterogeneity above this level will be interpreted with caution, and we will check the magnitude and direction of effects and the strength of evidence for heterogeneity from the *p* value of the chi^2^ test. If appropriate, the pooled result will not be reported.

We will also consider separately different group categories of surgery types (i.e. knee replacement, knee arthroplasty), as part of heterogeneity exploration.

#### Assessment of reporting bias

If there are more than 10 studies in our meta-analysis, we will explore potential publication bias by a funnel plot.

#### Data synthesis

Where studies are sufficiently homogeneous, we will carry out meta-analysis as per the Cochrane handbook of Systematic Reviews of Interventions using Review Manager 5.3 (RevMan 2014) [20].

We expect that pain will be reported in a number of different pain scales, and hence, the pooled SMD and 95% CI will be estimated using the inverse variance method. We will report the pooled results by back-transforming it into the (0-10) visual analogue scale by multiplying the SMD and 95% CI by the standard deviation of the largest study control group reporting the outcome of interest.

For dichotomous data, the pooled RR will be calculated using the Mantel-Hanzel method along with the 95% CI.

Where meta-analysis is not possible, we will report findings as a narrative or table as appropriate.

As all corticosteroids (specifically glucocorticoids) are thought to act in the same way, they will be no distinction (or subgroups) in the analysis. Different dosages will be converted into oral prednisolone equivalents using standard steroid conversion charts [23]. Similarly for opiate consumption postoperatively, given they act on the same pathway, they will be standardised into oral morphine equivalents for comparative reasons using a standardised opiate conversion chart [24].

#### Subgroup analysis and investigation of heterogeneity

If data allows it, we will explore the following subgroups:

- Systemic corticosteroid (oral/iv) versus local corticosteroid (intra/periarticular) on acute post-operative pain.
- The effect of corticosteroid on post-operative pain in different types of knee surgery, i.e. arthroscopic versus joint replacement.
- Different adjuvant corticosteroid dosage on post-operative pain. This will be based on the converted prednisolone equivalent.

#### Sensitivity analysis

We will conduct sensitivity analysis based on blinding and allocation concealment domains of the risk of bias tool; high risk studies will be excluded from the analysis to see how robust the result is.

Specifically for quasi-randomised studies, if they are assessed as high risk of bias for the sequence generation and allocation concealment domains of the risk of bias tool, we will use sensitivity analysis to explore the effects on the pooled results.

#### Summary of findings

We will use the GRADE tool to assess the quality of evidence of the outcomes and will include the findings in a ‘Summary of findings’ (SOF) table according to the Cochrane recommendations [25, 26]. The following outcomes will be included in the SOF: mean pain scores at 12, 24, 48 and 72 h, opiate consumption over the first 24 h, nausea and vomiting over the first 24 h and infection rate.

## Discussion

This review will assess the benefits and harms of perioperative adjunctive use of corticosteroids in knee surgery. The multimodal analgesic approach has reduced the post-operative use of oral opiates, but there is room for improvement given 75% of patients still report inadequate pain relief in the immediate post-operative period.

Our preliminary searches have not demonstrated any obvious issues conducting the GRADE assessment in our review and that most studies are adequate sized with a sample size above 50 patients in total, with a range of *n* = 30 to *n* = 270.

However, we have identified that a few papers have not reported the CI/SE/SD for the pain scores reported. We will attempt to contact authors for this information. Failing this if the results are illustrated graphically, we will use Measure C software to extrapolate these readings. If this is not available, then whatever data is available will be reported narratively and not included in the meta-analysis.

Furthermore, although most studies report pain on the visual analogue scale, there are two papers that report pain on other scales. One reports the pain outcome on the verbal response scale and the other on the visual descriptor scale. Given that these cannot be converted into the visual analogue scale, these will be reported narratively but not included in the meta-analysis.

A couple of papers have reported the outcome pain as a median with an interquartile range. Again, this will be described narratively and not included in the meta-analysis.

One paper from our search was only available in Persian and will therefore need to be translated to extract the data of interest. All other papers are readily available in English.

Our preliminary search demonstrated year of publications from 1993 to 2016. Papers from the 1990s may prove difficult to contact the authors for further information given they were published a long time ago.

In conclusion, we do not anticipate any major concerns conducting this review and issues described above should be relatively straightforward to manage.

## Appendix 1

### Ovid Medline search strategy

1 Steroids/or HYDROCORTISONE/or BETAMETHASONE/or Prednisolone/or Dexamethasone/or Triamcinolone/or Cortisone/or Glucocorticoids/or Adrenal-Cortex-Hormones/or Corticosterone/or Hydrocortisone/or 18-Hydroxycorticosterone/or Tetrahydrocortisol/or Tetrahydrocortisone/or Desoxycorticosterone/or 18-Hydroxydesoxycorticosterone/

2 ("adrenal cortex hormon$" or steroid$ or corticosterone or corticosteroid$ or cortisol$ or hydrocortisone$ or 18-hydroxycorticosterone or cortison$ or tetrahydrocortisol or hydrocortison$ or betamethason$ or prednisolon$ or methylprednisolon$ or dexamethason$ or triamcinolon$ or deflazacort$).ti,ab.

3 1 or 2

4 Surgical Procedures, Operative/or Surgery/or Perioperative-Care/

5 (surg$ or operat$ or (perioperative adj3 care)).mp. [mp = title, abstract, original title, name of substance word, subject heading word, keyword heading word, protocol supplementary concept word, rare disease supplementary concept word, unique identifier]

6 4 or 5

7 Pain, Postoperative/

8 ((postoperative adj4 pain*) or (post-operative adj4 pain*) or post-operative-pain* or (post* adj4 pain*) or (postoperative adj4 analgesi*) or (post-operative adj4 analgesi*) or "post-operative analgesi*").mp.

9 ((post-surgical adj4 pain*) or ("post surgical" adj4 pain*) or (post-surgery adj4 pain*)).mp.

10 ("pain-relief after surg*" or "pain following surg*" or "pain control after").mp.

11 (("post surg*" or post-surg*) and (pain* or discomfort)).mp.

12 ((pain* adj4 "after surg*") or (pain* adj4 "after operat*") or (pain* adj4 "follow* operat*") or (pain* adj4 "follow* surg*")).mp.

13 ((analgesi* adj4 "after surg*") or (analgesi* adj4 "after operat*") or (analgesi* adj4 "follow* operat*") or (analgesi* adj4 "follow* surg*")).mp.

14 7 or 8 or 9 or 10 or 11 or 12 or 13

15 (randomised controlled trial or controlled clinical trial).pt. or randomised.ab. or placebo.ab. or clinical trials as topic.sh. or randomly.ab. or trial.ti.

16 3 and 6 and 14 and 15

### EMBASE search strategy

1 steroid/or hydrocortisone/or betamethasone/or prednisolone/or dexamethasone/or triamcinolone/or cortisone/or glucocorticoid/or corticosterone/or hydrocortisone/or 18-hydroxycorticosterone/or tetrahydrocortisol/or tetrahydrocortisone/or deoxycorticosterone/or 18-hydroxydeoxycorticosterone/

2 (adrenal cortex hormon$ or steroid$ or corticosterone or corticosteroid$ or cortisol$ or hydrocortisone$ or 18-hydroxycorticosterone or cortison$ or tetrahydrocortisol or hydrocortison$ or betamethason$ or prednisolon$ or methylprednisolon$ or dexamethason$ or triamcinolon$ or deflazacort$).ti,ab.

3 1 or 2

4 surgical-technique/or surgery/or perioperative-period/or (surg$ or operat$ or (perioperative adj3 care)).ti,ab.

5 Pain, Postoperative/

6 ((postoperative adj4 pain*) or (post-operative adj4 pain*) or post-operative-pain* or (post* adj4 pain*) or (postoperative adj4 analgesi*) or (post-operative adj4 analgesi*) or "post-operative analgesi*").mp.

7 ((post-surgical adj4 pain*) or ("post surgical" adj4 pain*) or (post-surgery adj4 pain*)).mp.

8 ("pain-relief after surg*" or "pain following surg*" or "pain control after").mp.

9 (("post surg*" or post-surg*) and (pain* or discomfort)).mp.

10 ((pain* adj4 "after surg*") or (pain* adj4 "after operat*") or (pain* adj4 "follow* operat*") or (pain* adj4 "follow* surg*")).mp.

11 ((analgesi* adj4 "after surg*") or (analgesi* adj4 "after operat*") or (analgesi* adj4 "follow* operat*") or (analgesi* adj4 "follow* surg*")).mp.

12 5 or 6 or 7 or 8 or 9 or 10 or 11

13 3 and 4 and 12

14 (placebo.sh. or controlled study.ab. or random*.ti,ab. or trial*.ti,ab.) and human*.ec,hw,fs.

15 13 and 14

16 1 or 2

17 4 and 12 and 14 and 16

### CENTRAL search strategy

ID Search Hits

#1 MeSH descriptor: [Steroids] explode all trees

#2 MeSH descriptor: [Hydrocortisone] explode all trees

#3 MeSH descriptor: [Betamethasone] explode all trees

#4 MeSH descriptor: [Prednisolone] explode all trees

#5 MeSH descriptor: [Dexamethasone] explode all trees

#6 MeSH descriptor: [Triamcinolone] explode all trees

#7 MeSH descriptor: [Cortisone] explode all trees

#8 MeSH descriptor: [Glucocorticoids] explode all trees

#9 MeSH descriptor: [Adrenal Cortex Hormones] explode all trees

#10 MeSH descriptor: [Corticosterone] explode all trees

#11 MeSH descriptor: [Hydrocortisone] explode all trees

#12 MeSH descriptor: [18-Hydroxycorticosterone] explode all trees

#13 MeSH descriptor: [Tetrahydrocortisol] explode all trees

#14 MeSH descriptor: [Tetrahydrocortisone] explode all trees

#15 MeSH descriptor: [Desoxycorticosterone] explode all trees

#16 MeSH descriptor: [18-Hydroxydesoxycorticosterone] explode all trees

#17 (adrenal cortex hormon* or steroid* or corticosterone or corticosteroid* or cortisol* or hydrocortisone* or 18-hydroxycorticosterone or cortison* or tetrahydrocortisol or hydrocortison* or betamethason* or prednisolon* or methylprednisolon* or dexamethason* or triamcinolon* or deflazacort*)

#18 (#1 or #2 or #3 or #4 or #5 or #6 or #7 or #8 or #9 or #10 or #11 or #12 or #13 or #14 or #15 or #16 or #17)

#19 MeSH descriptor: [Surgical Procedures, Operative] explode all trees

#20 MeSH descriptor: [General Surgery] explode all trees

#21 MeSH descriptor: [Perioperative Care] explode all trees

#22 (surg* or operat* or (perioperative near care)):ti,ab

#23 (#19 or #20 or #21 or #22)

#24 MeSH descriptor: [Pain, Postoperative] explode all trees

#25 ((postoperative near/4 pain*) or (post-operative near/4 pain*) or post-operative-pain* or (post* near/4 pain*) or (postoperative near/4 analgesi*) or (post-operative near/4 analgesi*) or ("post-operative analgesi*")):ti,ab,kw

#26 ((post-surgical near/4 pain*) or ("post surgical" near/4 pain*) or (post-surgery near/4 pain*)):ti,ab,kw

#27 ("pain-relief after surg*" or "pain following surg*" or "pain control after"):ti,ab,kw

#28 (("post surg*" or post-surg*) and (pain* or discomfort)):ti,ab,kw

#29 ((pain* near/4 "after surg*") or (pain* near/4 "after operat*") or (pain* near/4 "follow* operat*") or (pain* near/4 "follow* surg*")):ti,ab,kw

#30 ((analgesi* near/4 "after surg*") or (analgesi* near/4 "after operat*") or (analgesi* near/4 "follow* operat*") or (analgesi* near/4 "follow* surg*")):ti,ab,kw

#31 #24 or #25 or #26 or #27 or #28 or #29 or #30

#32 #18 and #23 and #31

## Additional file

Additional file 1: [PRISMA P CHECKLIST AS PER JOURNAL GUIDANCE] (DOCX 33 kb)

## Abbreviations

CI: Confidence interval
PONV: Post-operative nausea and vomiting
RCT: Randomised controlled trial
SD: Standard deviation
SE: Standard error
SMD: Standardised mean difference
SOF: Summary of findings
